# Service User and Service Provider Perceptions of Enablers and Barriers for Refugee and Asylum-Seeking Women Accessing and Engaging with Perinatal Mental Health Care Services in the WHO European Region: A Scoping Review Protocol

**DOI:** 10.3390/ijerph19020937

**Published:** 2022-01-14

**Authors:** Kathleen Markey, Anne MacFarlane, Maria Noonan, Mairead Moloney, Susann Huschke, Kate O’Donnell, Claire O’Donnell, Teresa Tuohy, Ahmed Hassan Mohamed, Owen Doody

**Affiliations:** 1 Department of Nursing and Midwifery, Faculty of Education and Health Sciences, Health Research Institute, University of Limerick, V94 T9PX Limerick, Ireland; maria.noonan@ul.ie (M.N.); mairead.moloney@ul.ie (M.M.); claire.odonnell@ul.ie (C.O.); Teresa.G.Tuohy@ul.ie (T.T.); owen.doody@ul.ie (O.D.); 2 School of Medicine, University of Limerick, V94 T9PX Limerick, Ireland; anne.macfarlane@ul.ie (A.M.); susann.huschke@ul.ie (S.H.); 3 School of Medicine, Dentistry & Nursing, University of Glasgow, Glasgow G12 9LX, UK; 4 Community Sponsorship Support, Doras, Central Buildings, V94 W275 Limerick, Ireland; a.mohamed@doras.org

**Keywords:** perinatal mental health care, access and engagement with services, WHO European region, refugee and asylum-seeking women, scoping review, protocol, candidacy framework

## Abstract

There is a need to understand the specific perinatal mental health care needs of migrant subgroups who often have differing health care needs and specific barriers to accessing and engaging with health care services. It is important to have evidence about the WHO European context given the rising numbers of refugees and asylum seekers in the region. The aim of this scoping review is to map the factors that enable and prevent access and engagement of refugee and asylum-seeking women with perinatal mental health care services in the WHO European Region, from the perspectives of service providers and service users. The database search will include PsycINFO, Cochrane, Web of Science, MEDLINE, EMBASE, CINAHL complete, Scopus, Academic Search Complete, and Maternity and Infant Care (OVID). Search results will be exported to an online tool that provides a platform to help manage the review process, including title, abstract, and full-text screening and voting by reviewers independently. Data concerning access and engagement with health care services will be mapped on to the candidacy framework. Systematically searching evidence within the WHO European region and examining this evidence through the candidacy lens will help develop a more comprehensive and a deeper conceptual understanding of the barriers and levers of access and engagement with perinatal mental health care services, whilst identifying gaps in existing evidence. Exploring factors that influence access and engagement for refugee and asylum-seeking women from the perspective of key stakeholders in the service provision and/or service utilisation of perinatal mental health care services will add a more comprehensive understanding of the recursive relationship between service provision and use.

## 1. Introduction

With expanding globalisation and growing migration trends within Europe, health care organisations are challenged with providing optimal health care that meets the needs of refugees and asylum seekers. In 2019, the European Union was the destination for 10% of the world’s refugee population [1]. The unique mobility experiences, migration trajectory and causes for migration for refugees and individuals seeking asylum increase their vulnerabilities to serious health concerns [2]. Consequently, the World Health Organisation Europe’s Strategy and Action Plan for Refugee and Migrant Health (2016–2021) accentuates the importance of adapting health care services to meet the needs of refugee and migrant populations [3]. However, a recent scoping review examining the broad provision and access to general health care for refugees and migrants in Europe reported continued inequalities with access to services and unmet health care needs [4]. In particular, the high rates of maternal mortality in Europe among refugee and asylum-seeking women is a growing concern [5,6]. As evidenced by a recent review of systematic reviews on maternal care, there is growing evidence illuminating deficits in maternal care resulting in adverse pregnancy outcomes among women with asylum-seeking or refugee status [7]. Collectively, this evidence suggests the importance of critically reviewing strategies for providing culturally responsive health care services that are easily accessible and meet the unique needs of refugee and asylum-seeking women. Many factors influence a person’s decision to migrate from their country of origin or former habitual residence. Therefore, the importance of acknowledging the differing migrant typologies and appreciating that migrants are a heterogenous group of people is paramount. In the absence of a universally accepted definition of migrant, there are variations in definitions that are sometimes used interchangeably for different groups of migrants in different areas. Therefore, providing a standardised and credible definition of the migrant group that a research study is concerned with is essential. For the purposes of this scoping review, the United Nations High Commissioner for Refugees (UNHCR)’s definitions of refugees and asylum seekers is adopted [8]. A refugee is defined as ‘a person who owing to a well-founded fear of being persecuted for reasons of race, religion, nationality, membership of a particular social group, or political opinion, is outside the country of their nationality and is unable to or, owing to such fear, is unwilling to avail himself/herself of the protection of that country’ [8]. An asylum seeker is defined by the UNHCR as ‘an individual who has applied for asylum on the grounds of persecution in their home country relating to their race, religion, nationality, political belief or membership of a particular social group and remains classified as asylum seeking for as long as the asylum application is pending’ [8].

Perinatal mental illness is a common complication of pregnancy and the postpartum period, that encompasses a wide range of new or recurrent mental health conditions, ranging from anxiety and depression to more serious mental health conditions. Consequently, it is a growing public health concern that requires focussed attention. Refugee and asylum-seeking women are particularly vulnerable to perinatal mental health illness, due to the range of physical and psychological stressors experienced before, during or after migration [9,10,11,12]. The mounting evidence on the area of perinatal mental health among refugee and asylum-seeking women draws attention to the importance of planning targeted culturally responsive interventions and supports that are easily accessible and meet the needs of this marginalised group. However, this requires developing a comprehensive understanding of refugee and asylum-seeking women’s behaviours in seeking perinatal mental health support and how perinatal mental health care services are offered and navigated. This paper presents a protocol outlining how a scoping review will examine and map the existing evidence on service user and service provider perceptions of enablers and barriers for refugee and asylum-seeking women accessing perinatal mental health care, through a candidacy lens.

## 2. Background

The growing evidence reporting migrant women’s experiences of engagement with health care services across the globe identifies a range of challenges, which may prevent refugee and asylum-seeking women from seeking help and accessing perinatal mental health support. The various forms of social marginalisation that impact on perinatal mental health seeking behaviour [13], precarious migration status [14], communication [15] and cultural appropriate care [16,17], are commonly reported challenges experienced, that require attention. Although this body of evidence helps us understand specific care needs that require consideration, it does not comprehensively identify specific enablers and barriers to access and engagement with perinatal mental health care services. While some literature reviews focus on examining perinatal experiences of refugee women [18], most reviews predominantly focus on examining perinatal experiences of migrant women generally [10,17,19]. The term migrant that is adopted in many of the existing reviews in the area is an overarching term used that does not distinguish between migrant subgroups that often have differing health care needs and specific barriers to accessing and engaging with services that are sometimes unique to their subgroup. Although this body of evidence is helpful in understanding some of the complexities experienced by migrant women broadly during the perinatal period, combining heterogeneous migrant, asylum-seeking and refugee populations limits the depth of evidence. Differentiating between different migratory experiences and recognising the specific legal and social circumstances of women entering the asylum system/refugees is paramount to allow nuanced analyses and empirically grounded recommendations for policy and practice. Heslehurst et al. [7], in their detailed study of systematic reviews on the area of perinatal health care and outcomes among migrant populations, highlight the need to address limitations of reviews that combine heterogenous migrant, asylum-seeking and refugee populations. This scoping review will address this gap by focusing on evidence, which addresses factors that influence access and engagement with services for refugee and asylum-seeking woman, as a distinct group of people, who have unique needs influenced by their migration experiences.

Health care professionals play a critical role in supporting refugee and asylum-seeking women who are at risk of experiencing perinatal mental illness. In particular, their role in supporting access and engagement with perinatal mental health services is paramount. The importance of early identification and appropriate management of perinatal mental health conditions through screening, diagnosing and planning effective culturally responsive interventions during the antenatal and postnatal period is acknowledged in the literature. Viveiros and Darling [20] and Simpson et al. [21] draw attention to the challenges experienced by health care professionals when providing perinatal mental health care to women from diverse cultural and linguistic backgrounds. However, there is a need for a more wide-ranging understanding of the experiences of health care professionals and the role they play in supporting refugee and asylum-seeking women experiencing perinatal mental health illness. Thus, it is timely to comprehensively synthesise the evidence on various factors that enable and hinder access to/engagement with perinatal mental health care services for refugee and asylum-seeking women, in the WHO European region. Developing a comprehensive understanding of factors that influence access and engagement with perinatal mental health care services from the perspectives of key stakeholders (service utilisers and service providers) will add new insights into an area that to date has received fragmented attention.

Accessibility and engagement are multifaceted concepts and understanding these complex concepts within marginalised populations requires in depth inquiry. Examining the process of access and engagement with perinatal mental health care services among refugee and asylum-seeking women through the candidacy lens [22] will add to the knowledge base as it will provide a more comprehensive conceptual understanding of enablers and barriers for this marginalised group. Candidacy in this context is concerned with the way people seek health care, navigate health care services and the different stages of a person’s journey to health care. Candidacy describes access to health care services as a series of interactions between service users, health care professionals providing the service, and the health care system, thus providing a comprehensive understanding of factors that influence access and engagement with health care services. The candidacy framework consists of seven interlinking phases that provide an understanding of health seeking behaviour, how people navigate services and examine interactions between service user and service providers and recognises the impact of the wider context in which such interactions are played out. A unique component of the candidacy framework is that it highlights the importance of interactions between the service user and service provider. Candidacy has been used to better understand health care access for a range of populations and settings, including use of public sector services [23]; women’s experiences of domestic abuse [24]; symptom recognition and appraisal in chronic disease [25] and health care entitlement for asylum seekers in Canada [26]. This review will map its results onto the candidacy framework [22] as a means of developing a more comprehensive conceptual understanding of factors that influence refugee or asylum-seeking women access to/engagement with perinatal mental health care services, in the WHO European region.

## 3. Materials and Methods

### 3.1. Design

A scoping review methodology has been selected as it allows the bringing together of all relevant information not previously combined [27]. This is important given that literature is complex and heterogeneous. In addition, scoping reviews identify the nature of a concept and how that concept has been studied over time. To ensure rigour in our approach, this scoping review protocol outlines how the six-stage framework by Arksey and O’Malley [28] and extensions by Levac et al. [29], Peters et al. [27], Bradbury-Jones et al. [30] and Westphaln et al. [31], will be utilised. Scoping reviews are useful for systematically and broadly examining the scope and nature of existing evidence in a particular area, whilst identifying gaps in existing evidence as a means of informing future research. Data will be drawn together to achieve the aim of our review [32] and map the access and engagement barriers and enablers for refugee and asylum-seeking women. The scoping review will allow for the systematic mapping of the literature available, and identification of key concepts, theories, sources of evidence and gaps in the research [27]. The review, mapping and summarisation of the evidence will allow for the breadth or depth of the literature and knowledge gaps to be identified [33]. The Preferred Reporting Items for Systematic Reviews and Meta-Analyses Extension for Scoping Reviews (PRISMA-ScR) [33] will be used in the reporting of this review. This protocol will discuss how each of the methodological stages of undertaking a scoping review will be undertaken. The aim of this scoping review is to map service provider and service users’ perceptions of factors that influence access to and engagement with perinatal mental health care services for refugee and asylum-seeking women in the WHO European Region, through the candidacy lens.

Planning a well-structured review question that aligns to the overall objectives is essential in guiding the direction of the review [34]. Following a preliminary scoping of the literature to identify what reviews have been completed in the area, it was evident that there is a need to examine the scope and nature of the existing evidence around the area of access and engagement specifically for refugee and asylum-seeking women in the WHO European region. In line with recommendations by Pollock et al. [32], this scoping review question is broad in nature. The Population, Concept and Context (PCC) framework helped guide the formulation of the review question: What are service user and service providers’ perceptions of factors that enable and impede access to and engagement with perinatal mental health care services for refugee and asylum-seeking women in the WHO European Region?

### 3.2. Validity and Reliability

#### 3.2.1. Population

The scoping review will focus on perceptions of key stakeholders in the service provision and/or service utilisation of perinatal mental health care services for women with refugee or asylum-seeking status experiencing perinatal health conditions during pregnancy or 1 year postpartum.

#### 3.2.2. Concept

This review aims to capture factors that influence refugee and asylum-seeking women’s access to and engagement with perinatal mental health care services and supports provided. All evidence within the area of perinatal mental health—care, prevention, treatment, service provision and service utilisation—will be included and mapped onto the candidacy framework as a means of providing a deeper conceptual understanding.

#### 3.2.3. Context

All contexts where perinatal mental health care services are provided within the WHO European Union region will be considered eligible and included (e.g., home, hospitals, health care facilities, community, and support accommodation).

#### 3.2.4. Eligibility Criteria

Literature describing/detailing factors that influence refugee and asylum-seeking women’s access to/engagement with perinatal mental health care services and supports, from the perspective of service providers or service users, will be included. Studies and reports published within the WHO European Union region will be included, as Sweileh et al. [35] support the need for in-depth systematic reviews of literature by select migrant categories, by health domain (such as mental health), and by geographical demarcation. Studies and grey literature published in English since 1 January 2010, will be included.

Literature published in languages other than English, prior to 2010, referring to services outside the WHO European Union region, not focused on perinatal mental health or refugee and asylum-seeking women or do not differentiate their findings between migrant groups, even if they include refugees, will be excluded from this scoping review. While grey literature is included, literature reviews and non-primary research-based papers such as editorials, notes, letters, commentaries, discussion papers and opinion pieces will be excluded.

### 3.3. Literature Search Strategy

The proposed scoping review will utilise the six-stage framework by Arksey and O’Malley [28] and extensions by Levac et al. [29], Peters et al. [27], Bradbury-Jones et al. [30] and Westphaln et al. [31] to identify a wide range of information sources and ensure an inclusive approach to searching for relevant information to answer the review question. The search strategy has been developed by the team who have expertise in conducting reviews, searching databases, topic expertise and working with refugees and asylum seekers. A preliminary trial search was conducted in Scopus and CINAHL to pilot the search terms and identify if any scoping review in this specific area existed. This process was helpful in testing out search terms (keywords and subject headings) to determine if they return useful and relevant results in answering the review question and assisted in developing additional terms for concepts. The trial search identified inconsistencies with the use of terminology around refugee and asylum-seeking women. To address this, a comprehensive list of terms were developed (Table 1). Nine databases will be systematically searched: PsycINFO, Cochrane, Web of Science, MEDLINE, EMBASE, CINAHL complete, Scopus, Academic Search Complete and Maternity and Infant Care (OVID). Grey literature searches will incorporate guidance by Godin et al. [36] on systematically searching grey literature and identifying web based resources, which will include google scholar, Open Grey (http://www.opengrey.eu/, accessed on 12 December 2021), Grey Literature Report (https://greylit.org/, accessed on 12 December 2021), National Institute for Health and Care Excellence (https://www.nice.org.uk/About/What-we-do/Evidence-Services/Evidence-Search, accessed on 12 December 2021), Trip Medical Database (https://www.tripdatabase.com/, accessed on 12 December 2021) and the World Health Organisation Global Index Medicus (https://www.globalindexmedicus.net/, accessed on 12 December 2021). Pollock et al. [32] highlight the complexities with comprehensively searching grey literature. Therefore, for the purposes of this review the grey literature sourced will focus predominantly on published reports where screening will be carried out by title, executive summaries and contents pages when abstracts are not available, in line with guidance by Godin et al. [36]. The scoping review reporting methods outlined by the PRISMA-ScR scoping review extension [33] will be employed. The development of the search strategy will incorporate three phases: (1) initial search, (2) second search and (3) reference list search.

### 3.4. Study Selection, Data Extraction and Mapping

Study selection will be based on the eligibility criteria as outlined above. Search results will be exported to EndNote and duplicates removed. The de-duplicated results will then be imported to an online tool that provides a platform to help manage the review process and ensure transparency and rigour with screening. Each result will be voted on independently by at least two reviewers and any discrepancies or unresolved conflicts will be resolved through discussions within the team.

Following the screening process, papers that meet the eligibility criteria will proceed to the data extraction phase. The extracted data will align with the objectives of the scoping review and will include key information relevant to enablers and barriers for refugee and asylum-seeking women accessing and engaging with perinatal mental health services, from the perspective of both service users and service providers. The research team will collectively develop a data extraction table, which will be an iterative process where the data extraction template can be continuously updated as the reviewers become more familiar with the evidence. The initial variables selected for data extraction are informed by Pollock et al. [32] and are: (1) authors, year of publication, title and country of publication, (2) aims, objectives and research question (when applicable), (3) type of study, methodology, data collection and analysis methods, (4) service user and/or service provider population, characteristics, migration status (of service user), sample size, (5) definition of refugee or asylum seeker provided, (6) type and range of perinatal mental health care interventions/supports/services, (7) service user and/or service provider perceptions of factors influencing access to or engagement with (enablers/barriers) health care services, incorporating the candidacy framework, (8) summary of findings, (9) recommendations for future practice, policy and (10) limitations. A pilot test of this data extraction form with three papers will be carried out by two reviewers, which will inform amendments to the data extraction table as required. Data concerning access and engagement with health care services will be mapped to the candidacy framework [22].

The candidacy framework [22] incorporates seven ‘over-lapping stages’ involved in the process of seeking and accessing health care services. (1) Identification stage: data that refer to the point at which a refugee or asylum-seeking woman identifies themselves as needing help and a candidate for perinatal mental health services will be mapped to the identification stage of the framework. (2) Navigation stage: data that refer to the route to entry to perinatal mental health services for refugee and asylum-seeking women and the work that needs consideration in navigating the services will be mapped to the navigation stage of the framework. (3) Permeability stage: data pertaining to the ease at which a refugee or asylum-seeking woman can access a perinatal mental health service. (4) Presentation stage: data that refer to the act of asserting candidacy at a health service, either through an individual’s own decision or by an invitation. (5) Adjudication stage: data pertaining to the responses of health care professionals will be mapped onto the adjudication stage, which is the point at which health care professionals make a judgement on whether a refugee or asylum-seeking woman should be a candidate for perinatal mental health services. (6) Offers and resistance stage: data that refer to the point at which offers of care are made which may be accepted or rejected. The types of care offered and reasons for acceptance or rejection will be mapped on to the offers and resistance stage. (7) Operating conditions and local production of candidacy stage: data relating to the wider influences, including within the health system, on both refugee and asylum-seeking women and health care professionals that affect the production of candidacy will be mapped onto this stage.

Data will be collated, summarised and mapped by categorising aspects of evidence that report on factors that influence refugee and asylum-seeking women accessing and engaging with perinatal mental health care services. This review will map factors that influence access to and engagement with perinatal mental health care services for refugee and asylum-seeking women onto the seven overlapping stages of the candidacy framework [22]: (1) identification, (2) navigation, (3) permeability (4) presentation (5) adjudication (6) offers and resistance (7) operating conditions and local production of candidacy. The results will be presented in a diagrammatic and/or tabular form with an accompanying descriptive narrative synthesis to achieve the aims of the scoping review. Consideration will be given to the implications of the results in relation to the study purpose and potential implications that findings may have on future research, policy, and practice.

Consultation with knowledge users is an important stage of undertaking scoping reviews and adds to the methodological rigour of the review [29]. For the purposes of this review, this participatory consultation process will be iterative and continuous all the way through the review in different ways, as a means of providing further depth, context and meaning to the findings. The multidisciplinary review team consists of stakeholders from diverse backgrounds and particular expertise in the areas of migrant health, perinatal mental health, candidacy and evidence synthesis, including researchers with content expertise and representatives of community non-governmental organisations who have lived experiences of migration and support refugees and asylum seekers. The ranges of experiences and expertise within the review team provides a fruitful network for cross fertilisation of knowledge, skills, experiences and insights, which will ensure every step of the research process has relevance, meaning and utility and can be helpful in incorporating resources that may not emerge in database searches.

## 4. Discussion

This scoping review will provide an overview of the evidence that examines facilitators and barriers for refugee and asylum-seeking women accessing and engaging with perinatal mental health care services, in the WHO European region. It aims to build knowledge, enhance understanding and make recommendations around optimising access to and engagement with perinatal mental health care services for refugee and asylum-seeking women. A preliminary search of existing evidence identifies a paucity of evidence synthesis related to access and engagement with perinatal mental health care services for refugee and asylum-seeking women, from the perspectives of both service providers and service users. It also identified the need for research activities that address refugee and asylum-seeking woman as a distinct group of people and avoid heterogenous migrant research, which may not always be generalisable or appropriate. Within this distinct group are a subset of perinatal women with unique needs, which are further amplified by their personal migration experiences. This scoping review will add to this body of knowledge and address the need to further research in the area of perinatal mental health care among refugee and asylum-seeking women as a means of understanding specific access and engagement needs of different migrant subgroup populations in the WHO European region. The examination of evidence through a candidacy framework will support a deeper, more comprehensive and conceptual understanding of factors that influence access to and engagement with perinatal mental health care services for refugee and asylum-seeking women.

We envisage that the results of this scoping review which will be transparently reported using the PRISMA-ScR, will inform future research, practice and policy in the areas of perinatal mental health care supports for refugee and asylum-seeking women, in the WHO European region. The extensive pre-planning stage of this scoping review paid particular attention to approaches in ensuring methodological rigour. As a starting point, enlisting topical, methodological and database searching expertise within the review team, and incorporating a recommended scoping review methodology to inform the process, was critical. Collaboratively designing the scoping review protocol and planning the objectives, review question, eligibility criteria and how all the methodological steps of the review adhering to the scoping review framework will be completed helps the transparency and repeatability of procedures. Through publishing the research protocol, we strengthen the clarity of the search strategy, review process, reduce risk of bias and reduce the potential for unnecessary duplication of research.

## 5. Limitations

We acknowledge the limitation of inclusion of literature which is in English only, as this will mean that evidence published in languages other than English will not be included in the scoping review. No methodology quality appraisals of the documents included in the scoping review will be carried out, which is a potential limitation. However, carrying out quality appraisals of evidence is not the focus of scoping reviews as stated by Arksey and O’Malley [28] and re-affirmed by Pollock et al. [32]. Another limitation is that despite systematic searching, we may miss relevant papers, due to the diversity in terminology used and the nature of the concepts under review. Similarly, despite frequent consultation expected during study selection, it is possible that selection could be applied inconsistently, again related to the dispersed concepts and terminology. Heterogeneity makes it challenging to separate common elements, although analysis will be facilitated by use of the candidacy framework as a standardised template to code and map data.

## Figures and Tables

**Table 1:** Search strategy.

Terms for Refugee and Asylum Seekers	Terms for Perinatal Mental Health	Terms for Facilitators/Barriers Accessing/Engaging with Supports
1. Migrant * 2. Immigrant 3. Foreign * 4. Refugee * 5. Asylum * 6. Ethnic * 7. Minorit * 8. Race 9. Racial 10. BME 11. Nationality 12. Non-national 13. Non national 14. Displaced 15. Emigrant * 16. Non-native * 17. “Forced migration” 18. S1-S17 (OR)	19. Perinatal 20. Antenatal 21. Pregnancy 22. Childbirth 23. Postpar-tum 24. Postnatal 25. Maternal O 26. Postpartum 27. Pregnant * 28. Prenatal 29. Antepartum 30. “peripartum period” 31. “postpartum period” 32. Pre-natal 33. Peri-natal 34. Anti-natal 35. Peripartum 36. Post-partum 37. S19-S36 (0R) 38. Anxiety 39. Depression 40. Mental health 41. Mental illness * 42. Mental disorder * 43. Mood disorder * 44. PTSD 45. Post-traumatic stress disorder 46. Stress disorder 47. Psychiatric 48. Bipolar 49. Schizophrenia 50. Suicide * 51. Psychiatr * 52. Psychiatr * 53. Psycho * 54. Distress 55. “Mental illness” 56. “Mentally ill” 57. “Mental ill health” 58. Depress * 59. Trauma 60. Stress 61. S38–S60 (OR) 62. S37 AND S61	63. Experience * 64. Perception * 65. Know * 66. Inform * 67. Perspective * 68. View * 69. Believe * 70. Opinion * 71. Attitude 72. Idea * 73. Impression * 74. Uptake 75. Access 76. Use 77. Avail 78. Engag * 79. Behavior 80. Influenc * 81. Barrier 82. Facilitator 83. Hinder 84. Enable 85. Obstacle 86. “Help-seeking” 87. “Access to services” 88. “Service provision” 89. Services 90. Experience 91. S63–S90 (OR) 92. S18–S62–S91 (AND)

## Data Availability

Not applicable.
